# Junctional zone thickening: an endo-myometrial unit disorder

**DOI:** 10.52054/FVVO.15.4.109

**Published:** 2023-12-13

**Authors:** S Gordts, G Grimbizis, V Tanos, P Koninckx, R Campo

**Affiliations:** Leuven Institute for Fertility & Embryology, Schipvaartstraat 4, 3000 Leuven, Belgium; Aristotle University of Thessaloniki, 1st Dept Obstetrics and Gynaecology, Tsimiski 51 Street, 54623 Thessaloniki, Greece; University of Nicosia, Aretaeio Hospital, Dep Obstetrics & Gynaecology, Nicosia, Cyprus; Latifa Hospital, Dubai, United Arab Emirates, Prof. em. ObGyn KULeuven, Belgium; Life Expert Centre, Schipvaartstraat 4, 3000 Leuven, Belgium

**Keywords:** Adenomyosis, junctional zone, infertility, reproduction

## Abstract

Adenomyosis is a disease defined by histopathology, mostly of hysterectomy specimens, and classification is challenged by the disagreement of the histologic definition. With the introduction of Magnetic Resonance Imaging (MRI) and two- and three-dimensional ultrasound, the diagnosis of adenomyosis became a clinical entity. In MRI and US, adenomyosis ranges from thickening of the inner myometrium or junctional zone to nodular, cystic, or diffuse lesions involving the entire uterine wall, up to a well-circumscribed adenomyoma or a polypoid adenomyoma. The absence of an accepted classification and the vague and inconsistent terminology hamper basic and clinical research. The sub-endometrial halo seen at US and MRI is a distinct entity, differing from the outer myometrium by its increased nuclear density and vascular structure. The endometrium and the sub-endometrial muscularis or archimetra are of Müllarian origin, while the outer myometrium is non-Mullerian mesenchymal. The junctional zone (JZ) is important for uterine contractions, conception, implantation, and placentation. Thickening of the JZ can be considered inner myometrium adenomyosis, with or without endometrial invasion. Changes in the JZ should be considered a different entity than myometrial clinically associated with impaired conception, implantation, abnormal uterine bleeding, pelvic pain and obstetrical outcome. Pathology of the basal endometrium and JZ is a separate entity and should be identified as an endo-myometrial unit disorder (EMUD).

## Introduction

Adenomyosis is a disease defined by histopathology, mostly of hysterectomy specimens, the disagreement of the histologic definition challenges classification. The first description dates from 1860 (Rokitansky, 1860) when Karl Freiherr von Rokitansky described ‘fibrous tumours containing gland-like structures that resemble endometrial glands”. These structures were subsequently described as adenomyoma (Cullen, 1897; Cullen, 1919; Littlewood and Stewart, 1913), as endometriosis (Meighs, 1920), and later as adenomyosis (Frankl, 1925). The histological diagnosis of adenomyosis is defined as endometrial glands and stroma invading the myometrium deeper than 2.5 mm from the endometrial- myometrial junction with or without adjacent smooth muscle hyperplasia (Levgur et al., 2000). With the introduction of Magnetic Resonance Imaging (MRI) and two- or three-dimensional (2D, 3D) ultrasound (US), a non-invasive diagnosis of adenomyosis became clinically possible. Adenomyosis seen in MRI and/or US ranges from thickening of the inner myometrium or junctional zone (JZ) to nodular, cystic, or diffuse lesions in the uterine wall and well-delineated adenomyoma or a polypoid adenomyoma. The lack of an accepted classification and the vague and inconsistent terminology hamper basic and clinical research. Although JZ thickening and endometrial invasion of the myometrium are associated, it remains unclear whether these are two different pathologies, the JZ being specifically important for reproduction.

## What is known?

### Junctional zone imaging and characteristics

The immunohistochemical study from Noe et al. (1999) showed that the endometrium and the sub- endometrial muscularis exhibit a cyclic pattern of oestrogen receptors (ER) and progesterone receptors (PR) (Koninckx et al., 2022; Leyendecker et al., 2009); the outer myometrium is non-Mullerian since from mesenchymal origin and does not exhibit any cyclic changes. This archimyometrium is responsible for three types of uterine peristalsis (Kunz and Leyendecker, 2002): cervico-fundal, fundo-cervical, and isthmic peristalsis. Uterine peristalsis is important for early reproduction processes, such as sperm transport and high fundal implantation.

With the introduction of MRI, the inner myometrium or sub-endometrial layer of the myometrium, later called the JZ, was described as a low-intensity band between the endometrium and the myometrium (Hricak et al., 1983). Later, using the 3D US it was described as a sub-endometrial halo (Exacoustos et al., 2011). The sub-endometrial halo and the MRI image are distinct entities, differing from the outer myometrium by their increased nuclear density and vascular structure (Tetlow et al., 1999). Although the signal intensity on MRI shows a clear delineation with the outer myometrium, by histology, a strict zonation between the two entities cannot be identified (Harmsen et al., 2022; Mehasseb et al., 2011).

Using MRI, Brown et al. (1991) demonstrated that the JZ has a three-fold increase in nuclear density, a decreased extracellular matrix and lower water content. After trichrome histologic staining, transmission electron microscopy demonstrated that the JZ consists of an inner compact area and an outer transitional area, being part of the myometrium. The inner compact area runs parallel with the endometrium; beneath this area, the bundles of smooth muscles begin to take on a loosely organised arrangement, which becomes the transitional region of JZ. The myocytes in JZ differ from myocytes of the outer myometrium and have a higher proportion of nuclear area and a less closely packed extracellular matrix (Scoutt et al., 1991). Studying the morphometric features of the myometrium in pre-and postmenopausal women, with and without adenomyosis, Mehasseb et al. (2010) found clear differences between the inner myometrium and the outer myometrium, but the transition is gradual, with no distinct “zonation”. As such, the JZ is divided into a compact region and a transitional region. The compact region represents the hypointense MRI appearance of the JZ (Brown et al., 1991).

### Endo-myometrial unit

Human uterine glands develop during foetal growth and are completed at puberty (Cooke et al., 2013). The human endometrium has two zones: the stratum functionalis and stratum basalis. The stratum functionalis is shed during menstruation and regenerates from the underlying stratum basalis, resting on the sub-endometrial myometrium. Previously endometrial glands were considered single tubular structures terminating with a blunt end at the endometrial-myometrial junction (Gargett et al., 2016). Recently, endometrial glands have been shown to have a more complex structure (Tempest et al., 2022; Yamaguchi et al., 2021a; Yamaguchi et al., 2021b). 3D Immunohistochemistry revealed that the endometrial glands form a complex network in the stratum basalis expanding horizontally along the muscular layer, similar to the rhizome of grass. The “rhizome” of human endometrium has structural advantages as it may be more efficient than crypts in the preservation of epithelial stem/progenitor cells during the shedding of the endometrium. During menstruation, the plexus of the glands remains at the bottom of the endometrium. The rhizome structure is important for preserving the stem/progenitor cells, and the monoclonal endometrial gland will share genetic alterations of these stem progenitor cells to the several glands of the rhizome.

The three-dimensional morphologic structure of adenomyotic tissue showed the direct invasion of endometrial glands into the myometrium, with an ant colony network of ectopic endometrial glands in the myometrium (Yamaguchi et al., 2021a). This ant colony-like network of adenomyosis may represent the JZ disorder. It also was suggested that uterine peristalsis induced microscopic injuries in the endometrial-myometrial junctional zone and facilitated the transport of basal endometrium into the myometrium. Multiple “ultra-micro-ruptures” of the basal endometrial glands (stratum basalis) at various locations have been described using TEM (transmission electronic microscopy) (Ibrahim et al., 2015).

Myocytes of adenomyosis exhibit cellular hypertrophy and show differences in cytoplasmic organelles, nuclear structures, and intercellular junctions (Benagiano et al., 2012; Brosens et al., 2010). These myocytes likely facilitate the invasion of endometrial stromal cells (ESC) in comparison with controls, suggesting that adenomyosis is a disease of both the endometrial stroma and the myometrium (Mehasseb et al., 2010; Weimar et al., 2013). Recently the accumulation of M2 macrophages in adenomyosis was described, enhancing the invasion capacity of adenomyotic and healthy endometrial cells. Collective cell migration is supported by the presence of strong E- and N-cadherin-positive intracellular junctions in the basal glands (Stratopoulou et al., 2023).

Rasmussen et al. (2019b) compared junctional zone disease observed by ultrasonography and histopathology. By ultrasonography, three categories of JZ were described: (I) A linear JZ is a regular JZ without deep invagination of the endometrium, (II) a junctional zone disease (serrated junctional zone) with deep invagination of endometrium but still in contact with basal endometrium and (III) adenomyosis of the inner myometrium with deep invagination of the endometrium (≥2 mm), no longer in contact with the basal endometrium. The JZ expanded in both thickness and irregularity from linear JZmax and median JZdif (8.5 and 3.3 mm) to serrated JZ (10.1 and 4.1 mm) to adenomyosis of the inner myometrium (14.6 and 9.2 mm). Histopathology of a serrated JZ had a more than 3mm invasion of the endometrium without loss of contact with the basal endometrium. This serrated JZ had a JZmax of 8-12 mm. In 40.5% of the patients with ultrasonographic features of serrated JZ, adenomyosis, defined as the presence of endometrial glands and stroma in the myometrium without contact with the basal endometrium, could not be confirmed by histology. Most of the false positive cases had a serrated JZ. Ultrasonography (Exacoustos et al., 2011) showed that thickening and disruption of the JZ are strongly associated with uterine adenomyosis. However, by histology of the hysterectomy specimens, it was not clear that the JZ alterations seen on TVS were due to so-called “adenomyosis”.

### Junctional zone: imaging & histology

Histologic examination of the JZ, seen by TVS, suggests that the sub-endometrial halo is a distinct compartment of the myometrium comprising tightly packed muscle cells with increased vascularity (Tetlow et al., 1999). We (Gordts et al., 2008) did propose a classification with JZ hyperplasia as a separate entity with a thickness of 8 to 12 mm on T2-weighted images in women aged 35 years or less. This age limit was included as Hauth et al. (2007) indicated that the mean JZ width increases from 5 mm in women aged 20–30 years to 8 mm in those over 40 years. A JZ is described as normal when the appearance at MRI is regular, with a thickness of 5mm or less by TVS (Hauth et al., 2007). The term JZ hyperplasia was coined to define the thickening of the JZ in the absence of additional signs of adenomyosis (Brosens et al., 1995). Bird et al. (1972) described this as sub-basalis adenomyosis. By pathology on hysterectomy specimens, the most frequent menstrual disorders were not seen in patients with extensive adenomyosis but in women with sub- basal lesions.

The proliferation of the inner myometrial smooth muscle cells is not always linked to heterotopic endometrial implants, although considered an obligatory feature for the histologic diagnosis of adenomyosis. This confirms the findings of Rasmussen et al. (2019b), wherein the histology of the serrated JZ with a JZ max of 6-12 mm, no adenomyosis could be detected in 40% of the examined specimens. JZ thickening and focal lesions of the JZ must be interpreted carefully as changes in the JZ can be due to cyclic hormonal variations and to the thickening of the JZ by aging (Kunz and Leyendecker, 2002; Hauth et al., 2007).

Van den Bosch et al. (2015) published descriptive criteria developed through consensus under the acronym MUSA (Morphological Uterus Sonographic Assessment). Criteria were based on localisation, focal, diffuse, or cystic, lesion extent and size, and inner or outer myometrium involvement. Images of the JZ are described as regular, irregular, interrupted, and not visible in a coronal plane with hyperechogenic sub- endometrial lines or buds and hyperechogenic spots. A recent publication (Harmsen et al., 2022) described the discrepancies in JZ thickness seen at MRI and TVUS in the same patients with a thicker JZ if measured with MRI. JZ thickness was measured as one-third of the myometrium using MRI compared to a quarter using TVS, with the highest discrepancies seen at the luteal phase. The authors believed that the differences are due to the imaging capacity to visualise the external transitional area of the JZ and the change in tissue density.

The ultrasonographic appearances of echogenic sub-endometrial lines and buds, the irregular aspect, and the interruptions of the JZ can be considered expressions of the trauma caused by uterine dysperistalsis. As described by Leyendecker et al. (2015) “this uterine hyperperistalsis and myometrial contractions contribute to the uterine auto-traumatisation and therefore considered to be an important component of the TIAR process (Tissue injury and repair)”. Bright foci on MRI and TVUS hyperechogenic striae interrupted JZ, and buds should be considered direct signs of adenomyosis. Abnormal hysteroscopic findings are seen in these patients with endometrial changes like hyper-vascularisation, strawberry pattern, endometrial defects, and submucosal haemorrhagic cysts and these are all suggestive of adenomyosis (Puttemans et al., 2005; Molinas and Campo, 2006; Di Spiezio Sardo et al., 2017).

**Figure 1:**
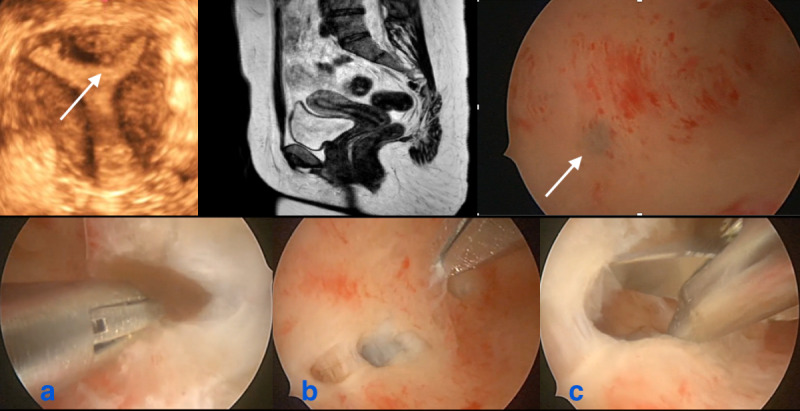
Indirect and direct images in the same patient: remark the small subendometrial cyst at ultrasound in the fundal area, also seen at hysteroscopy (white arrows), absent at MRI. Remark the normal aspect from the rest of the JZ. During the operative hysteroscopic procedure 4-5 small cystic structures, not identified with indirect imaging, were opened containing the typical brownish fluid of adenomyotic cysts. a: brownish fluid from small adenomyotic cyst; b: identification of another small cyst; c: insight view of the cyst.

Hysteroscopic exploration of the sub-endometrial myometrial layer provides pathognomonic signs of adenomyosis, such as neovascularisation and chocolate dye-filled cysts with endometrial implants on the pseudo-cystic wall (Di Spiezio Sardo et al., 2017). Hysteroscopy combined with ultrasound allows the exploration of the sub-endometrial zone and treatment of small adenomyotic cysts or tumours not always visualised by imaging (Gordts et al., 2018) (Figure 1). These hysteroscopic findings should be correlated with the results of US and / or MRI showing aberrations of the JZ (irregular, interrupted, sub-endometrial cysts) and histology.

Mavrelos et al. (2017) examined the impact of adenomyosis on the chance of clinical pregnancy after IVF. They remarked on the ultrasonographic features of adenomyosis as independent variables; the single feature associated with dysmenorrhea was the disrupted EMJ which was also the only feature associated with poor reproductive performance after IVF. Because of the association between the number of adenomyotic features at TVS and the severity of painful periods, a classification of the severity of adenomyosis based on the number of ultrasound features was suggested, with asymmetrical thickening and disruption of the EMJ associated with dysmenorrhea (Naftalin et al., 2016).

Adenomyosis is a dichotomous disease: it begins with an invagination of the endometrial basalis into the sub-endometrial layer and then infiltrates deeper into the myometrium (Brosens et al., 1995). NGS (new generation sequencing) supports the invagination theory in the pathogenesis of adenomyosis (Bulun et al., 2021). KRAS map mutations are limited to endometrial-type epithelial cells and were located in intracavitary endometrial tissue and proximally located adenomyotic lesions. Common aberrant gene expression patterns have been reported in endometriosis and adenomyosis, indicating a similar disease process in adenomyosis and endometriosis. Pre-existing epigenetics defects responsible for progesterone resistance, increased production of aromatase, and increased inflammation of the stromal cells in the basalis may increase the risk for the development of adenomyosis.

### Junctional zone and fertility

Maubon et al. (2010) reported the first paper on the impact of the thickening of the JZ on pregnancy rates after IVF. Implantation failures in patients with an AJZ (average JZ) >7mm are less than with an MJZ >10 mm (maximal JZ) (OR:45.39 by patient, OR 33.08 by transfer). The JZ thickness correlated with implantation failure at IVF, with an implantation failure rate of 95.8% for patients with a JZ zone between 7 mm and 10 mm, versus 37.5% in other patient groups (p<0.0001). Maged et al. (2017) confirmed that a thinner JZ at the time of OPU (ovum pick-up) was associated with a higher implantation rate. In patients with repeated IVF failures, pathological JZs are detected without more severe expressions of adenomyosis. Youm et al. (2011) found that an increased myometrial thickness without signs of adenomyosis, related most likely to a thickened JZ, was associated with lower birth rates. Clinical pregnancy rates were also lower in women with adenomyosis than in those without. The negative impact on IVF results was greater with adenomyosis localised in the inner myometrium and/or adjacent to the JZ (Iwasawa et al., 2021; Kobayashi et al., 2021). However, several publications did not find a negative impact of adenomyosis on IVF results. (Benaglia et al., 2014; Costello et al., 2011; Higgins et al., 2021).These conflicting results on implantation and pregnancy rates can be explained by the heterogeneity of the ovarian stimulation protocols and the lack of a detailed description of the adenomyotic lesion.

Without specifying the extent and/or localisation of the adenomyotic pathology, two meta-analyses confirmed the negative impact on pregnancy rates after IVF and an increased risk of miscarriage (Vercellini et al., 2014; Younes and Tulandi, 2017). A recent meta-analysis reported a lower live birth rate (LBR) (OR 0.59, 95% CI 0.37-0.92, p = 0.02), and clinical pregnancy rate (OR 0.66, 95% CI 0.48-0.90), with a higher miscarriage rate in women with adenomyosis (OR 2.11, 95% CI 1.33-3.33) (Cozzolino et al., 2022). The negative impact of diffuse adenomyosis appears to be more pronounced than the focal forms (Park et al., 2016). Ovulation induction protocols using hormonal suppression seem to counteract the negative impact of adenomyosis on pregnancy rates after IVF (Martínez-Conejero et al., 2011; Vercellini et al., 2014).

Recent data indicate that adenomyosis and endometriosis have a common endometrial dysfunction. Leyendecker et al. (2023) suggested the unifying denomination of archimetrosis for uterine adenomyosis and peritoneal and peripheral endometriosis as underlying pathogenesis of the evolution of the stratum vascular with repeated tissue injury and repair. Using MRI and hysterosalpingo-scintigraphy (HSSG), the latter allowing the observation of transport of labelled particles through the uterus, uterine adenomyosis and endometriosis were linked to hyper-peristaltic and dysperistaltic uterine activity with reduced fertility (Kissler et al., 2006). The hyperperistaltic constitution of the adenomyotic uterus impairs embryo implantation; a stepwise decrease in clinical pregnancy rates from the lowest to the highest uterine contraction frequency was reported (Fanchin et al., 1998). JZ, with an increased sub- endometrial halo, and associated with endometriosis (Kunz et al., 2000), was suggested to be caused by an infiltration of the endometrium and thus adenomyosis. It seems, therefore, that changes in junctional zone appearance and thickness, with or without adenomyosis, are associated with impaired implantation.

### Junctional zone and obstetric outcome

The JZ plays a critical role in deep placentation. In normal pregnancies spiral arteries in the JZ become greatly dilated by dynamic changes in the myometrial JZ, enabling adequate blood supply to the growing foetus. JZ pathology, as it is in patients with adenomyosis and endometriosis, fails to remodel the JZ segment of the spiral arteries. This incomplete transformation of the JZ spiral arteries results in defective deep placentation with a consequent variety of reproductive disorders. It was suggested that the primary site of vascular pathology in pregnancy is not in the placenta or decidua but in the JZ (Brosens et al., 2010). The endometrium and the inner uterine layer (endometrium sub-endometrium unit) are integral components of placentation and contribute to adverse obstetric outcomes.

Observational studies suggest that perturbances of the JZ before or at the moment of conception predispose to defective deep placentation (Arias et al., 1997; Tanos et al., 2019). Adenomyosis carries a 1.84-fold (95% CI 1.32-4.31, P=0.012) increased risk for preterm delivery and a 1.98-fold (95% CI 1.39-3.15, P=0.017) increased risk of PPROM (Juang et al., 2007a). These findings were confirmed in a more recent meta-analysis where adenomyosis was also significantly associated with an increased risk of pre-eclampsia, preterm delivery, small for gestational age infancy, and post-partum haemorrhage, which was confirmed after correction for age and mode of conception (Nirgianakis et al., 2021).

Another study reported the link between primary severe dysmenorrhea and the increased risk of preterm delivery (Juang et al., 2007b). As dysmenorrhea is one of the important clinical symptoms of adenomyosis, one could put hypothetically that a possible underlying existence of adenomyosis was present in these patients. This is important in adolescents with primary dysmenorrhea, mostly due to endometriosis. Adenomyosis is not only a pathology of adult life but was present in adolescents, and dysmenorrhea was the most reported symptom in 88% (Exacoustos et al., 2022; Martire et al., 2023). In a recent observational study, (Martire et al. 2023) evaluating the ultrasound findings in women with severe dysmenorrhea aged 12-25 years, reported an incidence of adenomyosis of 5.1% in the group 12-16 y, 14.9% between 17-20y and 22.3% between 21-25y. Further research can evaluate if, in the presence of early signs of JZ alterations, hormonal medication will be able to alter the further development of the disease.

#### Our hypothesis

With the generalised use of imaging, all abnormalities of the JZ have been described as indicative of adenomyosis. However, we suggest that abnormalities of the JZ should be distinguished from more advanced forms of adenomyosis. Discussing whether these abnormalities of the sub- endometrial layer were separate entities, Tocci et al. (2008) proposed to consider the “endometrial– sub endometrial myometrium unit (or junctional zone myometrium) disruption disease” as a new entity distinguished from adenomyosis.

2D - 3D ultrasound and MRI allow mapping of the endometrium, sub-endometrial or junctional zone, and outer myometrium as distinct zones. This allows the detection of early changes possibly related to adenomyosis. Differences in imaging of the JZ by MRI and US in comparison with the outer myometrium are due to an increased nuclear density and vascularity. Moreover, ontogenetically the inner and outer myometrium are of different origins.

The JZ plays is importance for conception, implantation, and placentation; dysfunction is associated with infertility, repeat miscarriage, impaired obstetric outcome, dysmenorrhea, and abnormal uterine bleeding. The endo-myometrial area presents ultra-structurally, a separate unit with endometrium spreading like a rhizome into the adjacent myometrium, a structure having advantages for the preservation of stem-progenitor cells and endometrial regeneration. However, alterations in the endometrial cells also can spread easily in the entire endo-myometrial area.

Thickening of the JZ detected by imaging reflects changes in smooth muscle organisation and differs from the important features of adenomyosis (presence of ectopic endometrial glands) which can account for discrepancies between imaging and histology (Gordts et al., 2008). Thickened JZ was repeatedly reported as to impair fertility, often without fitting the histological diagnosis of adenomyosis. Thickening is not always associated with heterotopic endometrial implants and can be present without other expressions of adenomyosis on ultrasound or MRI. This is confirmed by the presence of small subendometrial cystic structures or subendometrial adenomas or other suspicious lesions on hysteroscopy in otherwise normal appearance of the JZ (Figure 1). Being a dichotomous disease, the presence of subendometrial microcystic structures and irregularities of the JZ on TVUS with the presence of interruptions, striae, and buds could be early signs of adenomyosis.

As mentioned above, incidence of adenomyosis in adolescents with severe dysmenorrhea is between 5-23 %. As dysmenorrhea is an expression of a uterine malfunction, it would be of interest to pay more attention to the presence of JZ pathology in these adolescents looking for early diagnosis and possible hormonal treatment with oestro- progestogens.

We hypothesise that the pathological changes in the JZ, whether hereditary or caused by repetitive trauma and repair (TIAR), with or without histologically proven adenomyosis, should be considered a different entity. This specific pathology of the basal endometrium and JZ could be specified as an endo-myometrial unit disorder (EMUD).

## Conclusion

A major problem in evaluating the impact of adenomyosis on reproduction is the heterogeneity of the different forms under the general term of “adenomyosis” mostly without specifying the phenotypical presentation of the disease. We hypothesise that the pathological changes in the JZ, considered as the direct signs of adenomyosis, whether hereditary or caused by repetitive trauma and repair (TIAR), with or without histologically proven adenomyosis, should be considered as a different entity. The importance of considering JZ changes as reflecting the inherent predisposition makes it easy to understand the association with specific changes in the Mullerian cells. More specifically it might explain that associated infertility also reflects this predisposition or inherited defect. This is important since it still is unclear whether infertility is a consequence of adenomyosis/ endometriosis or whether both are the consequence of a common (inherited) disorder. This is similar to the endometriosis/adenomyosis-associated disorders of pregnancy, which do not change after surgery for severe endometriosis. It also explains that severe dysmenorrhea occurs from the first menstruation onwards. Even more important is that understanding the mechanisms might result in the prevention of adenomyosis or endometriosis. Defining this specific pathology of the basal endometrium and junctional as an endo-myometrial unit disorder (EMUD) will offer the opportunity for a better evaluation and understanding of the impact of this pathology separately from the other forms of adenomyosis. Further research is needed to corroborate this hypothesis and a first line of research could be the investigation of the JZ in prepubertal girls from women with no versus those with severe hereditary risk, probably by using elastography or MRI.
